# Luteal Phase in Assisted Reproductive Technology

**DOI:** 10.3389/frph.2020.595183

**Published:** 2020-12-07

**Authors:** Jan Tesarik, Cristina Conde-López, Maribel Galán-Lázaro, Raquel Mendoza-Tesarik

**Affiliations:** MARGen Clinic, Granada, Spain

**Keywords:** luteal phase, assisted reproduction technology (ART), corpus luteum, progesterone, uterine receptivity, decidualization of endometrium, progesterone-induced blocking factor (PIBF), immune tolerance

## Abstract

Luteal phase (LP) is the period of time beginning shortly after ovulation and ending either with luteolysis, shortly before menstrual bleeding, or with the establishment of pregnancy. During the LP, the corpus luteum (CL) secretes progesterone and some other hormones that are essential to prepare the uterus for implantation and further development of the embryo, the function known as uterine receptivity. LP deficiency (LPD) can occur when the secretory activity of the CL is deficient, but also in cases of normal CL function, where it is caused by a defective endometrial response to normal levels of progesterone. LPD is particularly frequent in treatments using assisted reproductive technology (ART). Controlled ovarian stimulation usually aims to obtain the highest number possible of good-quality oocytes and requires the use of gonadotropin-releasing hormone (GnRH) analogs, to prevent premature ovulation, as well as an ovulation trigger to achieve timed final oocyte maturation. Altogether, these treatments suppress pituitary secretion of luteinizing hormone (LH), required for the formation and early activity of the CL. In addition to problems of endometrial receptivity for embryos, LPD also leads to dysfunction of the local uterine immune system, with an increased risk of embryo rejection, abnormally high uterine contractility, and restriction of uterine blood flow. There are two alternatives of LPD prevention: a direct administration of exogenous progesterone to restore the physiological progesterone serum concentration independently of the CL function, on the one hand, and treatments aimed to stimulate the CL activity so as to increase endogenous progesterone production, on the other hand. In case of pregnancy, some kind of LP support is often needed until the luteal–placental shift occurs. If LPD is caused by defective response of the endometrium and uterine immune cells to normal concentrations of progesterone, a still poorly defined condition, symptomatic treatments are the only available solution currently available.

## Introduction

Luteal phase (LP) is the period of time between the transformation of the dominant ovarian follicle into the corpus luteum (CL), shortly after ovulation, and either the establishment of pregnancy or the onset of menstrual bleeding (1). During the LP, the CL secretes progesterone and some other hormones that are vital for maintaining the endometrium in a condition favorable for embryo implantation and its further development (2). The CL secretory activity is maintained by pulsatile secretion of luteinizing hormone (LH) from the pituitary gland (1). However, each CL has a programmed life span beyond in which LH support is not sufficient for its maintenance (3, 4). Hence, in the absence of pregnancy, LP is terminated by a loss of functional and structural integrity of the CL, referred to as luteolysis (1). If pregnancy is established, the functional life span of the CL is extended by a process termed rescue of the CL (5). This process requires sufficient quantities of human chorionic gonadotropin (HCG) to be secreted by the early implanted embryo (6).

LP deficiency (LPD) refers to a situation where the secretory activity of the CL is impaired, but it can also appear in cases of a normal output of hormones from the CL, where it is caused by a defective endometrial and immune system response (7). Though originally described (8) and recently confirmed (7, 9) in natural ovulatory cycles, LPD is particularly frequent in assisted reproductive treatment (ART) attempts using controlled ovarian stimulation protocols (10, 11), leading to a need for LP support.

In this review, we will analyze the physiological basis of CL formation and maintenance, the roles of CL secretion for the establishment of pregnancy, the effects of different ovarian stimulation protocols on CL function, and the therapeutic possibilities of substituting for defective CL secretion and restoring the threatened CL function in the context of ART treatments.

## Physiological Basis of Corpus Luteum Formation and Maintenance

The formation of the CL is a direct consequence of the pre-ovulatory surge of pituitary LH, which acts through a protein kinase A pathway (2). The LH surge leads to the transformation of granulosa and theca interna cells to granulosa-lutein and theca-lutein cells, respectively. These steroidogenic cells collaborate with non-steroidogenic (endothelial, immune, and fibroblast) cells, all of which are essential to the synthesis and secretion of steroids (12). Ongoing increased LH levels, following the initial LH surge, are critical to the maintenance of the CL structural and functional integrity (1) until, in the case of pregnancy, this function is taken on by HCG secreted by the early implanted embryo (6, 13). This shift, referred to as rescue of the CL (5, 6), marks the end of the LP, but the CL will continue to cover the basic needs of the uterus for progesterone until its function is resumed by the placenta, a phenomenon called luteal–placental shift.

## Role of Corpus Luteum in Early Pregnancy

The principal function of the CL is the secretion of progesterone needed for structural and functional transformations of the endometrium, known as the transition from the proliferative to secretory phase. This phenomenon involves structural and functional changes occurring in epithelial and stromal cells of the endometrium, elongation of terminal arterioles to the endometrial lumen, and a dramatic increase in the number of CD56–/CD16+ uterine natural killer (uNK) cells, which are believed to play a tolerizing role in maternal allorecognition of fetal trophoblasts, rather than cytotoxicity (14). All these changes are orchestrated by a variety of molecules and overall regulated by steroid hormones among which progesterone plays a pivotal role (15, 16). In addition to progesterone, different types of cells present in the CL also secrete estradiol, vascular endothelial growth factor (VEGF), endocrine gland-derived VEGF (EG-VEGF), and the cytokines interleukin-1 β (IL-1β and tumor necrosis factor-α (TNF-α). All of these minor CL secretory products were shown to be important for proper regulation of the secretion of progesterone, the main CL product (2).

## Etiology of Luteal Phase Deficiency

First described by Georgeanna Seagar Jones (17), well before the era of *in vitro* fertilization (IVF), LPD has later fallen into oblivion until relatively recently. While LPD appears to be more frequent than thought previously even in natural ovulatory cycles (9), it is particularly significant in assisted reproduction. LPD can be caused by two different conditions: first, impaired CL secretory activity and, second, abnormal response of the endometrium to normal concentrations of CL products.

### Impaired Corpus Luteum Secretory Activity

**Figure 1:**
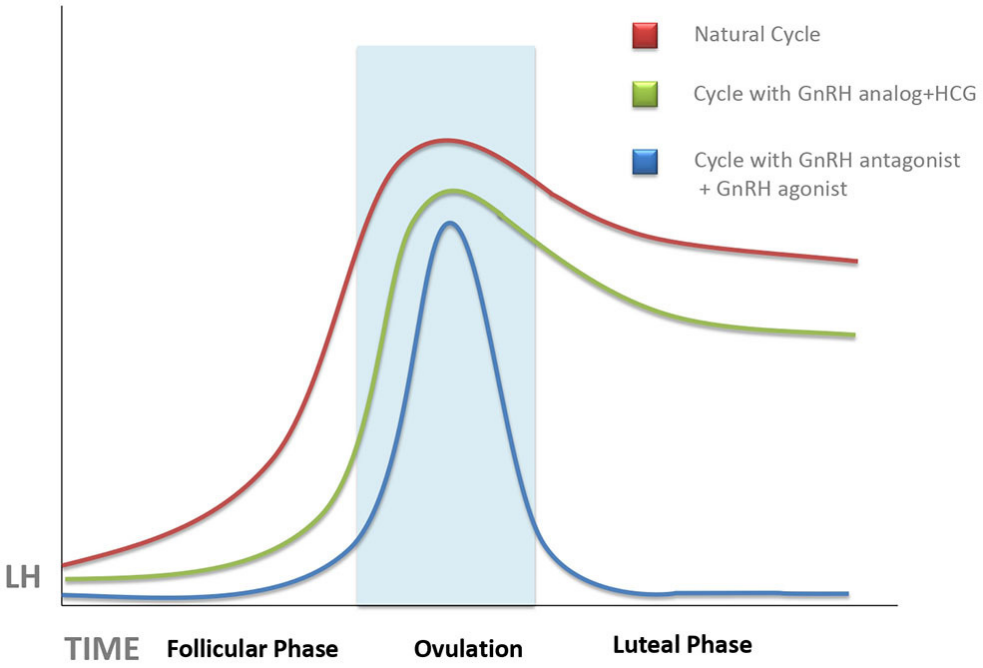
Schematic representation of the evolution of serum luteinizing hormone (LH) concentrations during a natural cycle (red line), after ovarian stimulation cycles triggered with human chorionic gonadotropin (HCG) (green line), and those trigged with gonadotropin-releasing hormone (GnRH) antagonist (blue line). Unless progesterone, HCG, or GnRH agonist is administered during the luteal phase, there is an abrupt fall of serum LH, which can provoke implantation failure. This tendency is more pronounced in GnRH agonist-triggered cycles as compared with HCG-triggered ones.

In most cases, impaired CL secretory activity is caused by inadequate stimulation by endogenous LH. This condition was already recognized in the early years of IVF (18–20). Initially, impaired CL secretory activity in IVF treatment attempts was attributed to the depletion of granulosa cells, precursors of the future granulosa lutein cells, during follicular aspiration (19), or to supraphysiologic steroid serum concentrations, routinely observed in stimulated cycles, affecting adversely LH secretion needed for CL formation, and maintenance (21). However, later studies pointed to the ovulation triggers, used to promote final oocyte maturation before performing follicular aspiration (Figure 1), or to the supraphysiologic steroid hormones secreted by the multiple CLs in the early LP of an IVF cycle, as the main culprit (22–24).

It has long been known that both estrogens and HCG suppress serum levels of LH, especially when both act together (25). Both actions are associated with conventional ovarian stimulation protocols in which multiple follicle growth leads to supraphysiologic estradiol levels, followed by the injection of HCG as ovulation trigger. In women treated by the long gonadotropin-releasing hormone (GnRH) agonist protocol, where pituitary GnRH release blockage is started in the cycle preceding ovarian stimulation, this deep pituitary suppression may persist after oocyte recovery and contribute further to deficient LH signaling in the forthcoming LP (26). Surprisingly, ovarian stimulation protocols using GnRH antagonists, instead of agonists, to prevent premature LH surge did not improve the situation (23), in spite of the fact that the GnRH antagonists, unlike the agonists, clear quickly and do not cause a long-term pituitary LH suppression (4).

The degree of LPD was further increased by the use of GnRH agonists, instead of HCG, as ovulation trigger in GnRH antagonist-controlled cycles (Figure 1). GnRH agonists have been used as ovulation trigger since the early 1990s (27, 28), but it was later discovered that GnRH agonist, when used as ovulation trigger, has a more powerful luteolytic effect than HCG (29). This was a limiting factor for the use of GnRH agonists as ovulation trigger in fresh embryo transfer cycles (except for oocyte donation) until it was discovered, in 2016, that increased luteolysis in GnRH agonist-triggered cycles can be prevented by continuous GnRH agonist administration throughout the LP, without a need for additional exogenous progesterone supplementation (30) as also confirmed by a recent meta-analysis (31). Dual and double triggers, combining a previous administration of GnRH agonist, followed by a small dose of HCG, were reported to improve oocyte quality without increasing the risk of severe ovarian hyperstimulation (32). However, the effects of these protocols on the LP remain largely unknown. It is of note that defective LP observed after agonist-triggered ovulation does not occur at random and is more pronounced in some women than in others; therefore, this patient-dependent response may be related to the personal characteristics of each patient's pre-ovulatory physiological surge profile (33).

### Abnormal Endometrial Response to Progesterone

In addition to low progesterone output from the CL, the clinical picture of LPD can also be caused by abnormal endometrial response to normal progesterone levels, a condition known as endometrial progesterone resistance. Most clinical data about progesterone resistance come from analyses of endometrial samples from patients suffering from endometriosis and polycystic ovary syndrome (PCOS).

Endometriosis (34) and PCOS (35) were the first pathological conditions in which progesterone resistance of human endometrium was discovered. In the case of endometriosis, the insensitivity to progesterone was suggested to be caused by chronic inflammation associated with this disease, making part of a vicious cycle whereby inflammation causes progesterone resistance, which further aggravates the inflammatory symptoms (36). As to PCOS, endometrial resistance to progesterone appears to be mainly caused by preferential expression of a less active progesterone receptor isoform in both epithelial and stromal cells of the endometrium (37).

Apart from endometriosis and PCOS, abnormal endometrial response to progesterone is also suspected to occur in some women lacking any of those pathologies, as evidenced by current experience with oocyte donation. In fact, recurrent implantation failure was observed after transfer of excellent-quality embryos originated from donated oocytes in spite of apparently normal endometrial proliferative phase and adequate serum progesterone concentrations after embryo transfer (38).

## Consequences of Luteal Phase Deficiency

During the LP, the endometrium undergoes a dynamic transition from proliferative to secretory morphology and function, a process orchestrated directly and indirectly by the sex steroids estrogen and progesterone and mediated by a complex array of secondary autocrine and paracrine factors including cytokines, chemokines, their receptors, and second messengers (39, 40). The timing of endometrial receptivity (implantation window) coincides with progesterone-induced downregulation of epithelial estrogen receptor alpha and with a shift in progesterone receptor out of the epithelial cells to the stromal compartment of the endometrium (41). According to some studies, this condition is associated with the appearance of endometrial epithelial pinopodes (42). Pinopodes, also called uterodomes, are smooth, membranous protuberances appearing on the apical surface of uterine epithelium when viewed under the scanning electron microscope. Reduction in the number, or an inappropriate time of maturation, of pinopodes was suggested to be associated with embryo implantation failure, although this conclusion has not yet been confirmed definitively. The role of the pinopodes is still under debate. They have been suggested to mediate pinocytosis and endocytosis of uterine fluid, thus facilitating adhesion of the blastocyst to the endometrium (43), to be directly involved in blastocyst–endometrial interaction through the expression of leukemia inhibitory factor (LIF) (44) or adhesion molecules, such as integrins (45), but all of these mechanisms still remain largely hypothetical (46).

Estrogen and progesterone affect endometrium through both genomic and non-genomic actions using different signal transduction pathways, and animal experiments show that selective deficiency of any of the second messengers and downstream signaling pathways can preclude correct endometrial response events in the presence of adequate concentrations of the hormones (47). It is thus conceivable that similar deficiencies can occur spontaneously in women and cause LPD even when estrogen and progesterone production is adequate. Little is known about the prevalence of these abnormalities in humans. Even though they are likely to be less frequent than the CL abnormalities, they have to be taken into consideration for designing the optimal strategy of LPD treatment in each individual patient.

**Figure 2:**
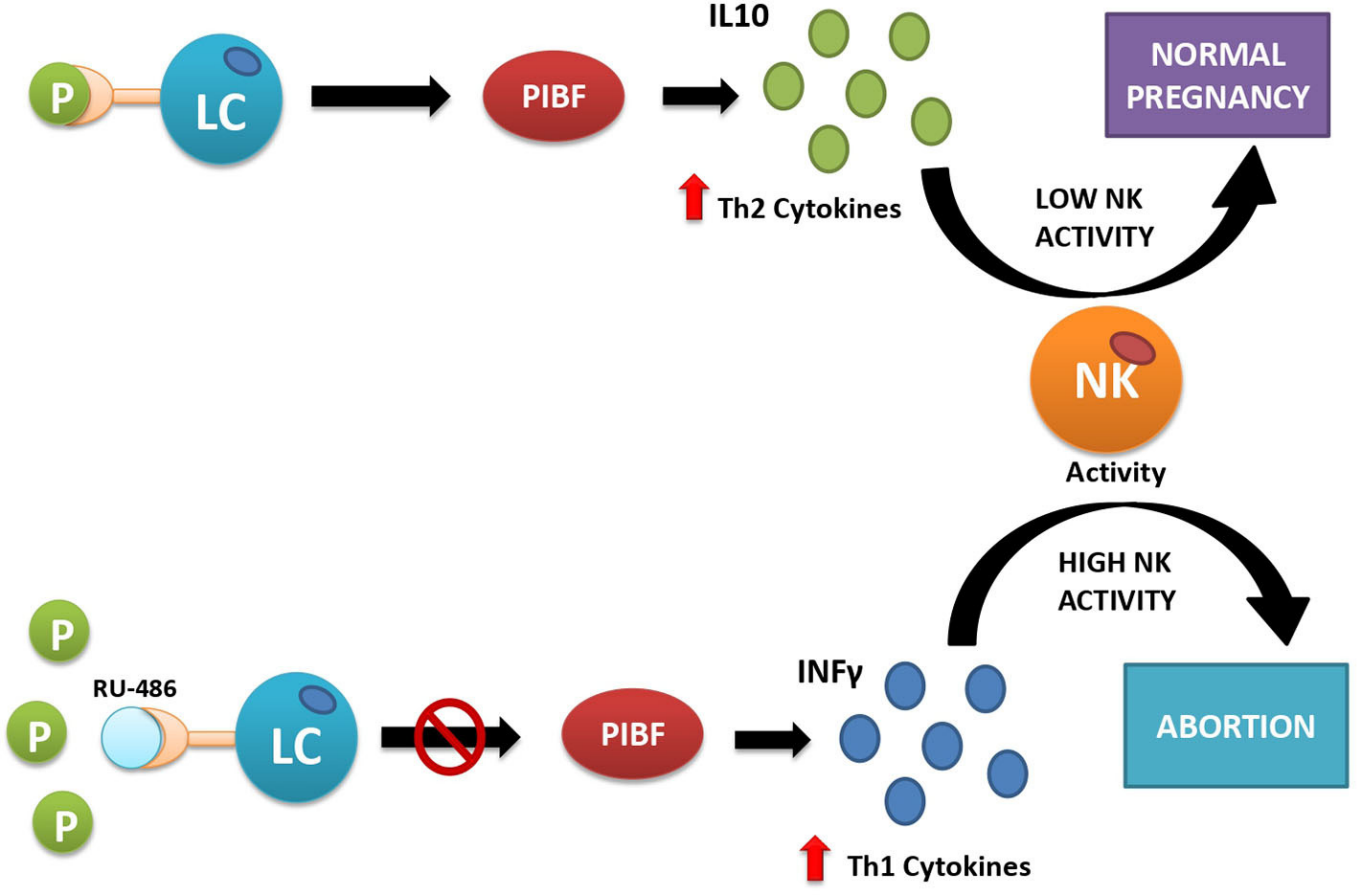
Interactions between progesterone and immune cells under normal conditions (upper part) and after artificially induced progesterone receptor insensitivity with the use of mifepristone (RU-486) (lower part). Under normal conditions (upper part of the figure), progesterone acts as its receptors located on the surface of some lymphocytes (LC). This action provokes the release of Th2-type interleukins (e.g., IL-10), which inhibit cytotoxic action of natural killer (NK) cells and facilitate embryo implantation. If the receptor is inactive, here simulated by blocking the progesterone receptor with the competitive antagonist RU-486 (lower part of the picture), the lymphocytes release Th1-type interleukins, e.g., interferon-γ (IFN-γ), which activate cytotoxic action of NK cells against the placental semiallograft, leading to abortion.

Apart from the disturbance of the molecular signaling underlying endometrium–blastocyst molecular crosstalk required for the blastocyst adhesion and invasion (48), progesterone secretion by the CL also has other biological functions that are essential for implantation and survival of the early implanted embryo. They include the role of progesterone in epithelial and stromal cell remodeling necessary for decidualization (49), moderation of uterine contractility after embryo transfer (50), and feto-maternal immunological crosstalk (51). This latter function of progesterone is mediated by a protein called progesterone-induced blocking factor (PIBF), synthesized by progesterone receptor-expressing lymphocytes and NK cells present both in the peripheral blood and in the decidua. The number of progesterone receptor-expressing lymphocytes increases throughout gestation, and it is significantly lower in women with recurrent miscarriages than in healthy pregnant women of corresponding gestational ages, suggesting a relationship between lymphocyte PR expression and the outcome of pregnancy (52). If PIBF is abnormally low as compared with progesterone, it may indicate relative lymphocytic insensitivity to progesterone (53). This condition creates a potential threat of implantation failure, abortion, and preterm delivery because of dominant Th1-type, pro-inflammatory cytokine production in response to the presence of the embryo, recognized by the immune system as a semiallograft (Figure 2). During normal pregnancy, in progesterone receptor-expressing lymphocytes, which represent 70% of decidual T cells (52), progesterone-activated receptor induces local secretion of Th2-type, anti-inflammatory cytokines that mediate the immunological tolerance of the embryo and promote its implantation and survival (52, 53). In particular, PIBF induces an increase in regulatory T cells (Tregs) and CD4+ CD25+ T cells whose role is to suppress the immune response (54). In addition to T cells, progesterone also affects uNK cells, a particular type of NK cells, different from those found in the peripheral blood (54, 55). During pregnancy, these cells lose their cytotoxic functions and play a supportive role by enhancing angiogenesis (43) and by dampening the activity of pro-inflammatory TH17 cells through the secretion of interferon-γ (56). They also inhibit the function of cytotoxic T cells through the expression of immunomodulatory molecules such as galectin-1 and glycodelin A (43).

## Management of Luteal Phase Deficiency in Assisted Reproductive Technology

The etiology of LPD associated with ART treatments has two components: the effect of ART procedures themselves and the individually variable inborn predisposition of each patient. Data show that virtually all women undergoing an ART attempt are prone to LPD (57, 58). It was hypothesized that women who experience LPD in natural cycles are exposed to a more severe LPD after ART treatment than women with normal spontaneous LP (9, 10). This relationship, however, still remains to be substantiated.

If the hypothesis of the relationship between LPD in natural and ART cycles was confirmed, it would be important to look for LPD before enrolling patients into an ART protocol. However, there persist serious doubts about whether, and how, LPD can be diagnosed reliably. The criteria used in the original description of LPD were based on the evaluation of basal body temperature charts (detecting a short LP), urine pregnanediol measurement, and a premenstrual endometrial biopsy (16). All of these three criteria have later been challenged (4). Serum progesterone concentration is known to fluctuate, following the rhythm of pituitary LH pulses (58). However, these fluctuations appear to be attenuated in cycles triggered with HCG and, in general, after treatment with ovarian stimulation medications (23). Thus, midluteal serum progesterone concentration still remains to be one of the most employed markers of LPD (59). More recent suggestions are based on uterine transcriptome analysis (46). A customized endometrial receptivity array, containing 238 genes related to endometrial receptivity (59), was suggested to be used in women with recurrent implantation failure (60). However, the utility of this method in the clinical practice has later been questioned (61). More recently, a smaller set of genes has been proposed to assess the receptivity status in biopsies obtained in the secretory phase (61).

### Luteal Phase Deficiency With Low Serum Progesterone Levels

Once LPD with low serum progesterone levels is diagnosed, the optimal treatment strategy has to be established individually in each case. In most cases, LPD can be avoided even without previous diagnosis, by meticulous control of the LP in each ART attempt. There are basically two types of LP support in ART procedures: one using exogenous progesterone preparations via different routes of administration to substitute for progesterone deficiency and the other aiming to stimulate endogenous progesterone production by the CL. The former strategy is feasible in all types of ART procedures, whereas the latter cannot be used in clinical scenarios using artificial LP after previous suppression of ovarian activity, such as most of the treatment attempts using frozen embryo transfer (FET) or donor oocyte cycles.

In the first years of ART history, the usual approach to be used to treat LPD was HCG treatment after embryo transfer (20). However, with the advent of more “aggressive” ovarian stimulation protocols, leading to the recovery of high number of oocytes, it was necessary to use HCG with caution, especially in cases of a high ovarian response, in order to reduce the risk of ovarian hyperstimulation syndrome (62). LP support was thus increasingly performed by a direct substitution of the missing endogenously produced progesterone with exogenous progesterone preparations administered by different routes. Progesterone for LP support can be administered orally, intramuscularly, vaginally, and, most recently, subcutaneously, with each route having different bioavailability and tolerability profiles (63). A recent meta-analysis (31) showed that intramuscularly and vaginally administered progesterone is equally effective, the latter being better supported by the patients. Recently, promising results were obtained with oral administration of dydrogesterone, a synthetic progestin, instead of progesterone for LP support (62). Dydrogesterone is a more patient-friendly treatment because of its oral administration. Moreover, it is not detected by the current laboratory tests for serum progesterone concentration, which makes it possible to detect the occurrence of luteal–placental shift of progesterone production and thus to determine the time from which LP support is no more required (64, 65).

More recent studies have revisited the idea of supporting the patient's own progesterone secretion by stimulating the CL activity. Instead of HCG, CL is stimulated by administration of GnRH agonists, which do not increase the risk of ovarian hyperstimulation syndrome, after embryo transfer (53). In addition to stimulating CL function, independently of the ovulation trigger used (30, 55, 66, 67), GnRH agonists also have a direct beneficial effect on viability of the implanting embryos (68).

### Luteal Phase Deficiency With Normal Serum Progesterone Levels

This form of LPD is quite difficult to diagnose because of the lack of reliable diagnostic tests demonstrating a failure of the endometrium to respond to normal levels of progesterone. Assays based on the analysis of endometrial transcriptome profile (59–61) may pave the route, but their interpretation is currently uncertain. Moreover, the transcriptome profile of endometrial cells will not reflect the deficiencies of progesterone-induced synthesis of PIBF by a subset of uterine progesterone receptor-expressing lymphocytes, a condition that can lead to the rejection of the embryo semiallograft (see *Consequences of Luteal Phase Deficiency*). Consequently, a question arises on how to treat repeated implantation failures and/or miscarriages with normal serum progesterone levels, some of which might be caused by uterine lymphocyte progesterone receptor failure.

Increasing serum progesterone concentration above the physiological levels does not appear to be a solution to this problem because abnormally high serum progesterone concentration can be harmful for endometrial receptivity and decidualization (69). There is currently little information about the prevalence of LPD with normal serum progesterone concentrations. In fact, such cases are likely to go undetected unless specific and not quite usual assays for the detection of the expression of the progesterone receptor and other players involved in the progesterone-activated signal transduction pathways are employed. Treatments used in these cases are thus largely symptomatic.

Rejection of the trophoblastic semiallograft by the maternal immune system can be treated by high doses of progesterone, but progesterone alone might not be sufficient. The inflammatory reaction, associated with the secretion of the pro-inflammatory Th1 cytokines, can be mitigated by high doses of melatonin, which also acts as a potent antioxidant agent (70). Due to these effects, melatonin promotes uterine and placental health and, consequently, favors embryo implantation and attenuates the risk of miscarriage (71). Vaginally administered sildenafil was also shown to mitigate maternal rejection of the implanted embryo and fetus (72), presumably by acting at the TNF-α level and modulating Treg and NK cell activity in women with recurrent pregnancy loss (73).

Vitamin D is another molecule that might be of help in women with partial insensitivity of their endometrium to progesterone (74). Indeed, both progesterone and vitamin D regulate the expression of the homeobox gene HOXA10, a molecule well-known to be involved in the mechanism of implantation, in human endometrial stromal cells (75) so that vitamin D might partly take on the role of progesterone in case of defective endometrial response mechanisms to this hormone. In addition, high doses of the anti-oxidant coenzyme Q10, previously shown to improve NK cell activity in patients with diabetes mellitus (76), may also be of help.

As to the lack of the attenuating effect of progesterone on uterine contractions provoked by embryo transfer, one solution might be postponing embryo transfer to day 5 of embryo development (7 days after ovulation trigger). It was reported that uterine contractility decreases progressively and reaches a nearly quiescent status 7 days after HCG administration, at the time of blastocyst transfers (77).

Recently, growth hormone has been shown to improve uterine receptivity in women with unexplained repeated implantation failure (78), including those in oocyte donation attempts with young oocyte donors, normal sperm characteristics, and fresh embryo transfer (79). The mechanism of this action is not known. However, growth hormone and progesterone share some components of their signaling pathways (46, 80, 81), so that the deficiency of one might be compensated by the other. Anyway, all this remains a pure, though stimulating, speculation unless future studies generate definitive answers to these issues.

## Conclusions

LPD can occur in natural ovulatory cycles, causing infertility, though the prevalence of this condition remains to be determined. In ART attempts, LPD is quite a frequent complication. It was speculated that LPD in ART cycles is caused by an overreaction of GnRH analogs (agonists or antagonists) used to prevent premature ovulation during ovarian stimulation and/or by the inhibitory action of the ovulation trigger (HCG or GnRH agonist) on the forthcoming LH secretion by the pituitary, responsible for the maintenance of the CL activity required for endometrial receptivity until its role is taken over by embryo-derived HCG. In addition to these causes of LPD, marked by a low midluteal serum progesterone concentration, other cases of LPD can be caused by a defective response of target cells (endometrial epithelial and stromal cells, and uterine T lymphocytes) to normal concentrations of progesterone. LPD can cause implantation failure and miscarriage through inappropriate endometrial preparation for implantation, embryo expulsion through uterine contractions after embryo transfer, or immune rejection due to a failure of progesterone-induced reprogramming of uterine T cells and NK cells. LPD caused by insufficient progesterone secretion by the CL can be easily corrected, either by hormonal stimulation of the CL or by a direct supplementation of exogenous progesterone. On the other hand, LPD caused by a defective response of target cells to normal progesterone stimulus is more difficult to diagnose. If suspected, its consequences have to be treated with the use of symptomatic therapeutic protocols.

The strength of this study is its broad coverage of the subject. The weakness is that many new data are still largely hypothetical and need further confirmation.

## Author Contributions

The concept of this review was designed by JT, MG-L, CC-L, and RM-T. The initial manuscript draft was undertaken by JT. CC-L, MG-L, and RM-T contributed substantially to manuscript revision. The figures were designed by JT, CC-L, and RM-T. All artwork was produced by CC-L.

## Conflict of Interest

The authors declare that the research was conducted in the absence of any commercial or financial relationships that could be construed as a potential conflict of interest.
